# ^**68**^Ga-PSMA-PET/CT for the evaluation of pulmonary metastases and opacities in patients with prostate cancer

**DOI:** 10.1186/s40644-018-0154-8

**Published:** 2018-05-16

**Authors:** Jonathan Damjanovic, Jan-Carlo Janssen, Christian Furth, Gerd Diederichs, Thula Walter, Holger Amthauer, Marcus R. Makowski

**Affiliations:** 1 Department of Radiology, Charité, Charitéplatz 1, 10117 Berlin, Germany; 2 Department of Nuclear Medicine, Charité, Augustenburger Platz 1, 13353 Berlin, Germany; 3 King’s College London, Division of Imaging Sciences, London, UK

**Keywords:** Lung metastasis, Pulmonary opacity, PSMA, PET/CT, Prostate cancer

## Abstract

**Background:**

The purpose of this study was to investigate the imaging properties of pulmonary metastases and benign opacities in ^68^Ga-PSMA positron emission tomography (PET) in patients with prostate cancer (PC).

**Methods:**

^68^Ga-PSMA-PET/CT scans of 739 PC patients available in our database were evaluated retrospectively for lung metastases and non-solid focal pulmonary opacities. Maximum standardized uptake values (SUV_max_) were assessed by two- and three-dimensional regions of interest (2D/3D ROI). Additionally CT features of the lesions, such as location, morphology and size were identified.

**Results:**

Ninety-one pulmonary metastases and fourteen opacities were identified in 34 PC patients. In total, 66 PSMA-positive (72.5%) and 25 PSMA-negative (27.5%) metastases were identified. The mean SUV_max_ of pulmonary opacities was 2.2±0.7 in 2D ROI and 2.4±0.8 in 3D ROI. The mean SUV_max_ of PSMA-positive pulmonary metastases was 4.5±2.7 in 2D ROI and in 4.7±2.9 in 3D ROI; this was significantly higher than the SUV_max_ of pulmonary opacities in both 2D and 3D ROI (*p*<0.001). The mean SUV_max_ of PSMA-negative metastases was 1.0±0.5 in 2D ROI and 1.0±0.4 in 3D ROI, and significantly lower than that of the pulmonary opacities (*p*<0.001). A significant (*p*<0.05) weak linear correlation between size and 3D SUV_max_ in lung metastases (ρ_Spearman_=0.207) was found.

**Conclusion:**

Based on the SUV_max_ in ^68^Ga-PSMA-PET alone, it was not possible to differentiate between pulmonary metastases and pulmonary opacities. The majority of lung metastases highly overexpressed PSMA, while a relevant number of metastases were PSMA-negative. Pulmonary opacities demonstrated a moderate tracer uptake, significantly lower than PSMA-positive lung metastases, yet significantly higher than PSMA-negative metastases.

## Background

Worldwide, prostate cancer (PC) is considered the second most frequently diagnosed cancer in men and the fifth leading cause of cancer death [1]. Radiolabeled prostate-specific membrane antigen (PSMA) ligands such as ^68^Ga-PSMA-HBED-CC have been introduced recently as promising radiotracers for the PET imaging of PC [2]. PSMA, or glutamate carboxypeptidase II, is a transmembrane protein expressed in the prostate as well as in many other tissues, and is significantly overexpressed in most prostate cancer cells [3]. Different studies have demonstrated the superiority of ^68^Ga-PSMA–PET imaging regarding the detection of metastases in PC, both compared to current standard imaging (CT, MRI and bone scintigraphy) and other PET tracers, such as ^18^F-Choline [4–7]. Especially at low serum PSA levels in biochemically recurrent prostate cancer, it improves the detection of metastatic lesions [8]. However, PSMA overexpression is not limited to prostate cancer; it is typically found in other malignant tumors, such as lung, colorectal, gastric, renal and thyroid cancer, particularly within the tumor neovasculature [9–12]. Furthermore, case studies have demonstrated elevated PSMA-expression in benign lesions such as sarcoidosis, Paget disease, meningioma and adrenal adenoma [13–16].

According to autopsy studies, pulmonary metastases are considered to be the second most common extranodal metastases in PC (46%), after bone metastases (90%) [17].

The clinical incidence of PC pulmonary metastases in a large retrospective review study has been 3.6% [18]. Although the data suggest that the presence of pulmonary metastases has no ominous impact on clinical course and disease outcome [19], early and reliable detection of lung metastases can be of high clinical importance for accurate staging and therapy planning.

Pyka et al. investigated the imaging and differentiation of pulmonary PC metastases, primary lung cancer and tuberculosis in 45 patients using ^68^Ga-PSMA–PET. Within their study population, SUV analysis was not able to differentiate pulmonary metastases from lung cancer [20]. Other studies have confirmed PSMA-overexpression in primary lung cancer [9, 21]. However, case reports have demonstrated PSMA-overexpression also in benign lung lesions such as pulmonary opacities and bronchiectasis [22], sarcoidosis [13] and tuberculosis [20].

Therefore, the aim of this study was to investigate the ^68^Ga-PSMA-PET imaging properties of lung metastases and opacities in PC patients, and whether quantitative SUV analysis is able to differentiate benign from malignant lesions.

## Methods

### Study population

For this retrospective study, we obtained approval from our institutional ethics review board. We extracted 739 consecutive patients with confirmed prostate cancer from our local database who underwent at least one ^68^Ga-PSMA-PET/CT between September 2013 and April 2017. By manual review of all reports and scans, we identified twenty patients with lung metastases and fourteen patients with pulmonary opacities, according to the criteria described below. Prostate cancer was histologically proven in all patients. Only patients with no other known type of cancer but PC were included. All available additional information from clinical records was considered.

### Positron Emission Tomography Tracer

Using a conventional ^68^Ge/^68^Ga radionuclide generator (Eckert & Ziegler Radiopharma GmbH, Berlin, Germany), ^68^Ga was eluted and then compounded with PSMA-HBED-CC (ABX GmbH, Radeberg, Germany) according to the method described previously [23, 24].

### Imaging protocol

PET/CT imaging was performed 67.0±33.1 min after intravenous injection of 125.9±26.9 MBq of ^68^Ga-PSMA-HBED-CC. PET scans were acquired using a Gemini Astonish TF 16 PET/CT scanner (Phillips Medical Systems) in 3D acquisition mode [25]. Axial, sagittal and coronal slices were reconstructed (144 voxels with 4mm^3^, isotropic). Prior to each PET scan, a CT was performed for anatomical mapping and attenuation correction (30 mAs, 120 kVp). Each bed position was acquired for 1.5 min with a 50% overlap. In nineteen patients, contrast-enhanced CT (CE-CT, 162 - 215 mAs, 120 kVp, slide thickness 3.0 mm) was performed using 70-120 ml of contrast agent (Ultravist® 370, Bayer Schering Pharma, Berlin, Germany), which was injected intravenously with a delay of 70 seconds for the venous phase. In fifteen patients, only unenhanced CT was available.

### Imaging analysis

Two experienced observers analyzed the PET/CT scans using Visage 7.1 (Visage Imaging GmbH, Berlin, Germany). All scans were reviewed for suggestive pulmonary lesions, and lesions were classified to either “metastases” or “opacities”. All the following criteria had to be fulfilled for the diagnosis of lung metastasis: (I) CT imaging with a singular or multiple masses. (II) New appearance or change of size of lesions compared to previous studies. (III) Lesions need to be round or oval. (IV) No signs of benignity such as fat or calcification. (V) Synchronous other distant metastases. For the diagnosis of pulmonary opacity, the following criteria had to be fulfilled: (I) CT-imaging of an irregular-shaped or confluent focal opacity. (II) Lesion must not be nodular or a solid mass. (III) Lesion must not be classified as metastasis. Only intrapulmonary lesions >5mm were considered. Patients with a history of or signs for a malignancy other than PC were excluded. Overall, 20 patients with lung metastases and fourteen patients with pulmonary opacities were identified. Up to ten lesions per patient were analyzed. In case a patient was imaged more than once, only the most recent ^68^Ga-PSMA-PET scan was included in this study. As a result, ninety-one lung metastases and fourteen pulmonary opacities were analyzed. For all lesions, location and morphology were described. The sizes of metastases were measured based on the CT scan.

Standardized uptake values (SUV) were normalized for body weight by the software using the equation *SUV* = *C*_*tis*_/*Q*_*inj*_/*BW*, where *C*_*tis*_ is the lesion activity concentration in MBq per milliliter, *Q*_*inj*_ is the activity injected in MBq, and *BW* is the bodyweight in kilograms. For PET data quantification, a two-dimensional region of interest (2D ROI) and a three-dimensional region of interest (3D ROI) were defined. ^68^Ga-PSMA-HBED-CC uptake of all lesions was quantified using maximum standardized uptake values (SUV_max_). To differentiate PSMA-positive from PSMA-negative lesions, the SUV_max_ of the blood pool measured within the descending thoracic aorta was set as a reference; lesions with SUV_max_-values 20% or more above blood pool were considered PSMA-positive. All values were recorded in the transaxial, attenuation-corrected PET-slice representing the greatest extent of the respective lesion. Regions of interest were defined avoiding the periphery of lesions to minimize partial volume effects. The readers were blinded to the results of other diagnostic procedures and the clinical history of the patients.

### Standard of reference

A reference standard was created by presenting each case to an adjudication panel consisting of experts in the fields of nuclear medicine, radiology and urology. All available data (clinical records and follow-up data, radionuclide imaging, radiographs, CT, MRI, histology, and intraoperative findings) were taken into consideration for the standard of reference and a final diagnosis for every lesion was documented.

### Statistical analysis

The descriptive statistics are reported as mean, median and/or range when applicable. The Mann-Whitney *U* test was used for the comparison of SUV_max_ values of pulmonary opacities and lung metastases. SUV_max_ values in 2D and 3D ROI were compared using the Wilcoxon signed-rank test. To determine the relationship between SUV_max_ and size of metastases, a Spearman’s rank correlation was used. The significance level was set to α < 0.05. Statistical analyses were conducted with SPSS 23 for Mac (IBM Corp, Armonk, NY).

## Results

### Characteristics of the study patients

In total, 91 lung metastases were detected in 20 of 739 (2.7%) patients and 14 pulmonary opacities were detected in 14 of 739 (1.9%) patients. Patients’ characteristics are summarized in Table 1. Mean patients’ age was 70.6±8.1 years. Median GS was 9 (range 6 – 10). Mean PSA level was 123.6±300.2 ng/ml.

**Table 1:** Characteristics of the study collective of PC patients with lung metastases. Summary of the patients’ characteristics, including age, PSA, GS and previous therapy. *GS* Gleason score, *PSA* prostate-specific antigen.

	Mean ± SD	Median (Range)	N (%)
Age (years)	70.6±8.1	71.7 (50.6 – 82.5)	
PSA (ng/ml)	123.6±300.2	10 (0.01 – 1423)	
Gleason score		9 (6 – 10)	
Therapy			
RP			17 (65.4)
RT			10 (38.5)
ADT			17 (65.4)
ADT ongoing			14 (53.8)
CTX			5 (19.2)

*RP* Radical prostatectomy, *RT* Radiotherapy, *ADT* Androgen deprivation therapy, *CTX* Chemotherapy

### Lesion-based analysis of pulmonary metastases and opacities

**Fig. 1:**
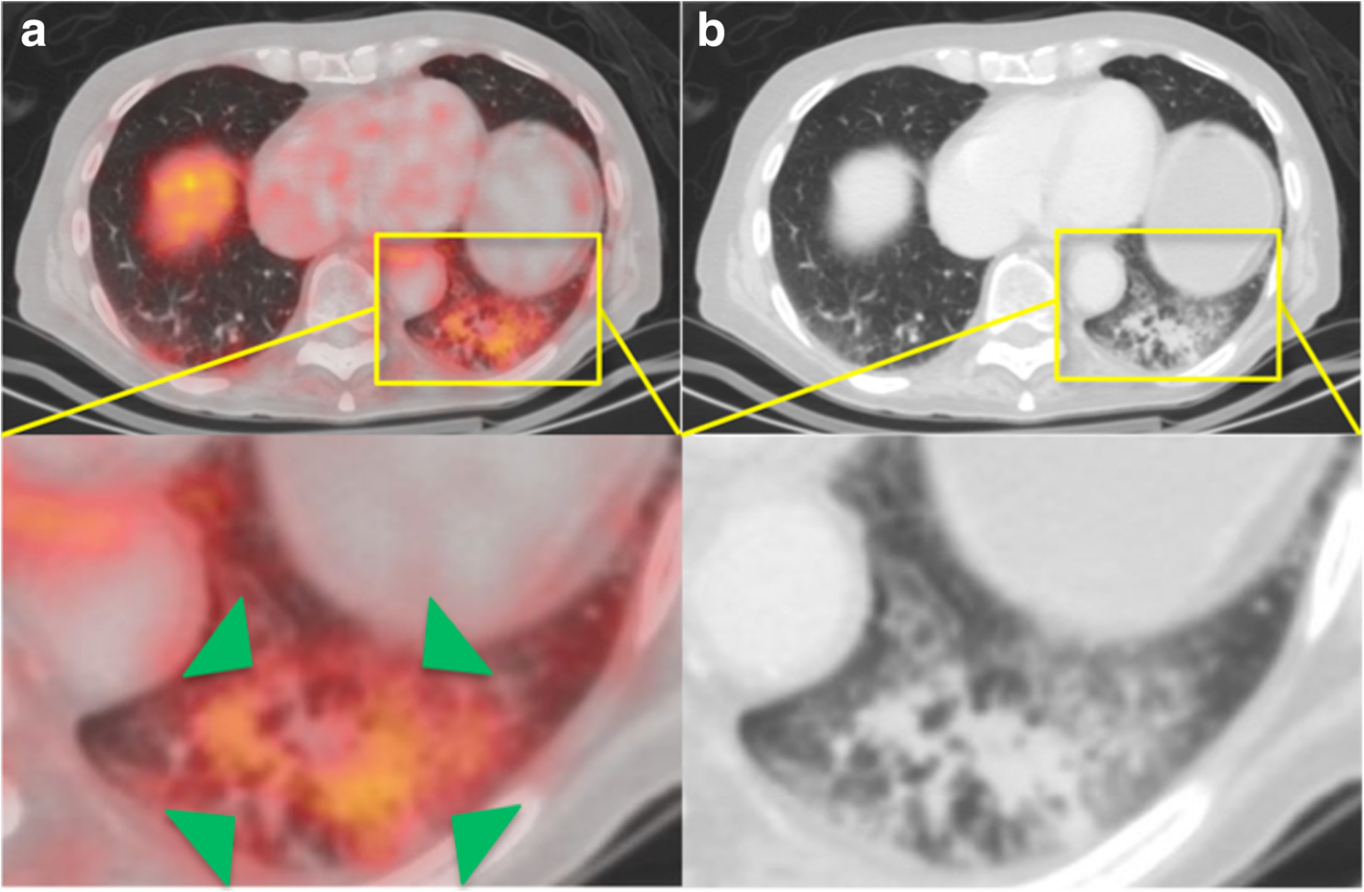
Example of pulmonary opacities in a PC patient. **a**, **b**: ^68^Ga-PSMA-PET/CT of an 82-year-old patient with an adenocarcinoma of the prostate. At initial diagnosis in 2010, the GS was 3+4. He had received androgen deprivation therapy only. The serum PSA was 6.48 ng/ml at the time of examination. PET/CT (**a)** revealed PSMA-positive peribronchial opacities, with SUV_max_-values up to 3.8 (green arrows). In CE-CT (**b**), the opacities appear as dense multifocal peribronchial opacifications and ground-glass-opacities, with partial confluence. It was interpreted as bronchopneumonia. *GS* Gleason score, *PSA* prostate-specific antigen, *SUV*_*max*_ Maximum standardized uptake value

**Fig. 2:**
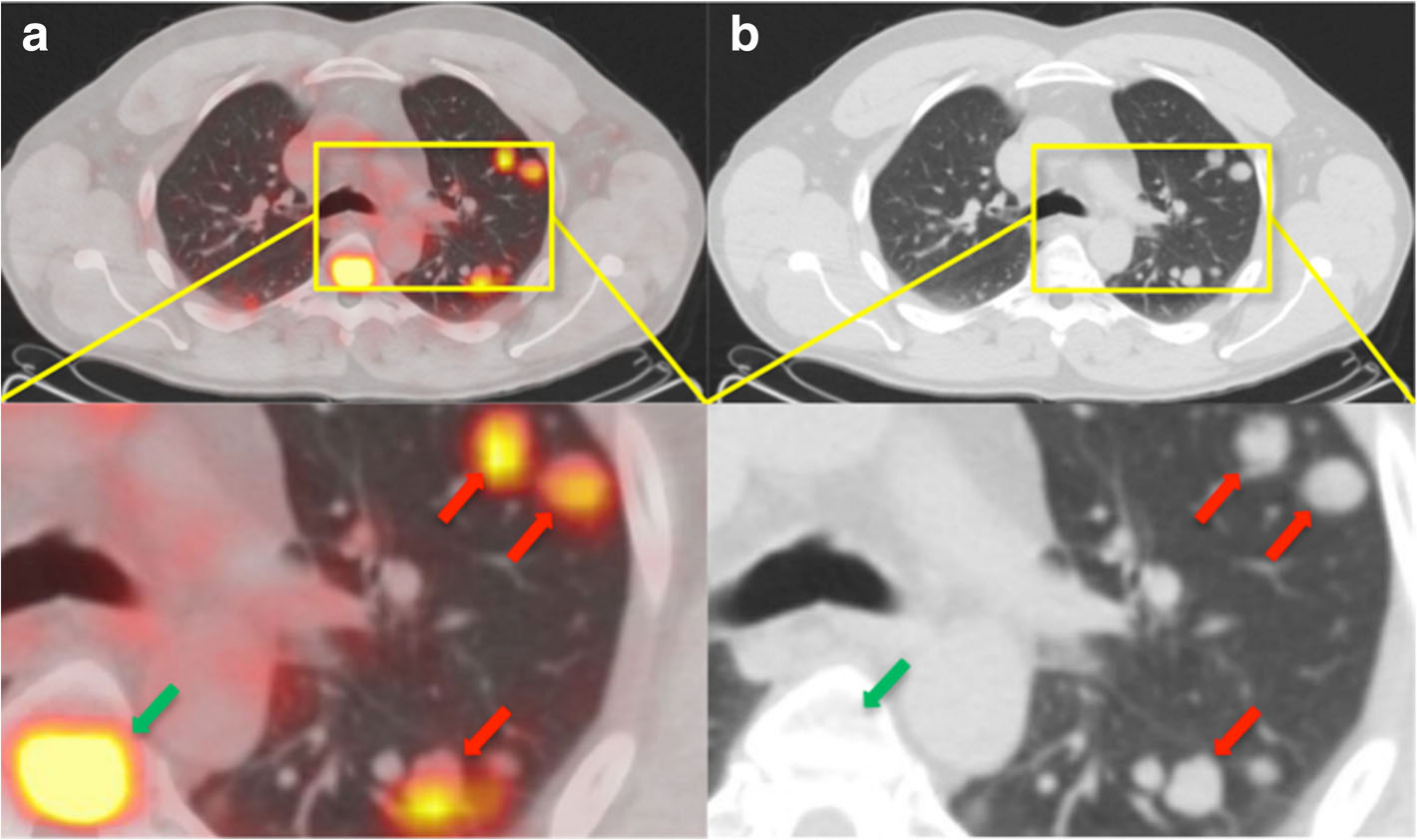
Example of ^68^Ga-PSMA-positive lung metastases in a PC patient. **a**, **b**: ^68^Ga-PSMA-PET/CT of a 50-year-old patient with a recurrent acinar adenocarcinoma of the prostate and lung metastases. After the initial diagnosis in 2013, he had received a radical retropubic prostatectomy followed by androgen deprivation therapy. The initial GS was 4+4. PET/CT (**a**) was performed because of PSA-persistency after the surgery and illustrates disseminated, PSMA-positive lung metastases, with SUV_max_-values up to 5.0. Red arrows point to examples of lung metastases in segments 1, 2 and 3. Besides, two spinal bone metastases und a residual tumor in the prostatic fossa were found. In unenhanced CT (**b**), lung metastases appear as dense, well-circumscribed, rounded lesions. *GS* Gleason score, *PSA* prostate-specific antigen, *SUV*_*max*_ Maximum standardized uptake value

**Fig. 3:**
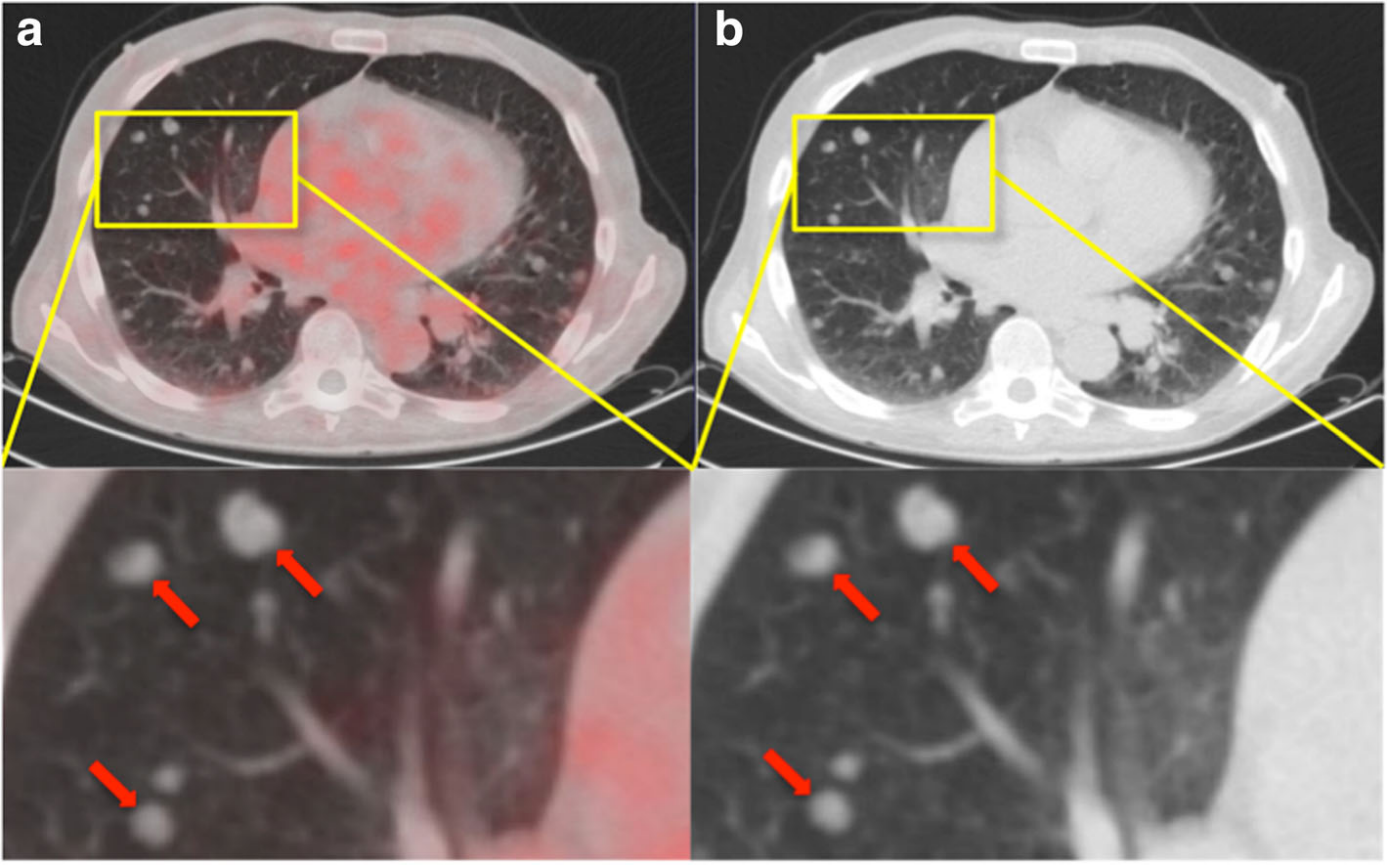
Example of ^68^Ga-PSMA-negative lung metastases in a PC patient with a partially neuroendocrine prostate carcinoma. a, b: ^68^Ga-PSMA-PET/CT of a 71-year-old patient with an acinar adenocarcinoma carcinoma of the prostate with partial neuroendocrine transdifferentiation and pulmonary, hepatic, osseous, and lymph node metastases. He had received a palliative prostatectomy and a radio- and chemotherapy. The initial GS was 4+5. The PET/CT (**a**) illustrates disseminated, PSMA-negative lung metastases, with SUV_max_-values from 0.35 to 1.75. Red arrows point to examples of lung metastases in segment 4. In unenhanced CT (**b**), lung metastases appear as dense, well-circumscribed, rounded lesions. *GS* Gleason score, *SUV*_*max*_ Maximum standardized uptake value

**Fig. 4:**
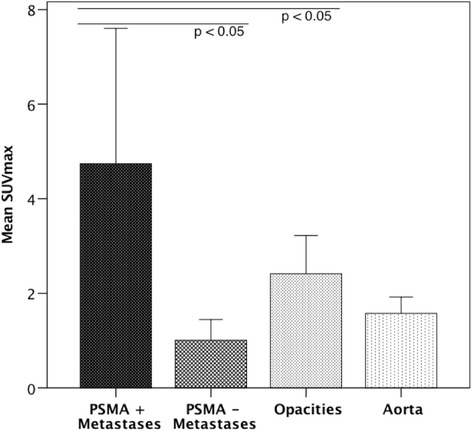
SUV_max_ of PSMA-positive and PSMA-negative lung metastases, pulmonary opacities and the aorta. Bar chart comparing the mean SUV_max_-values of PSMA-positive and PSMA-negative pulmonary metastases, pulmonary opacities and the aorta. The mean SUV_max_ of PSMA-positive lung metastases was significantly higher than that of pulmonary opacities (*p*<0.05). Contrary, the mean SUV_max_ of PSMA-negative lung metastases was significantly lower than that of pulmonary opacities (*p*<0.05). Error bars represent the standard deviation. *SUV*_*max*_ Maximum standardized uptake value

**Fig. 5:**
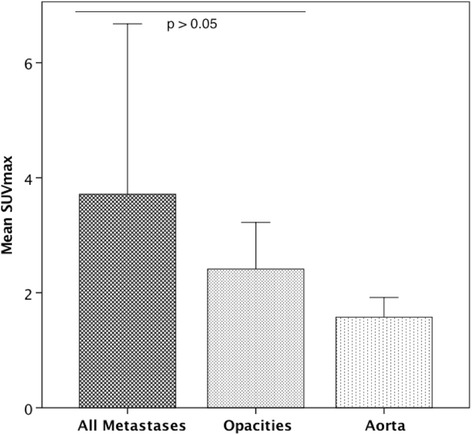
Mean SUV_max_ of all lung metastases, pulmonary opacities and the aorta. Bar chart comparing the mean SUV_max_-values of all pulmonary metastases, pulmonary opacities and the aorta. There was no difference between the mean SUV_max_ of all lung metastases compared to that of pulmonary opacities (*p*>0.05). Error bars represent the standard deviation. *SUV*_*max*_ Maximum standardized uptake value

The lesions’ characteristics such as location and morphology are summarized in Table 2, all detailed results in Table 3. The mean size of metastases was 11.0±6.3cm^2^ (range 0.2 – 29.5cm^2^). The mean SUV_max_ of all lung metastases was 3.5±2.8 in 2D and 3.7±3.0 in 3D ROI. In total, 66 PSMA-positive (72.5%) and 25 PSMA-negative (27.5%) metastases were identified. No significant difference regarding the size of metastases was measured between both groups. Examples of pulmonary opacities are illustrated in Fig. 1, examples of PSMA-positive and PSMA-negative metastases in Figs. 2 and 3. The mean SUV_max_ of PSMA-positive metastases was 4.5±2.7 in 2D and 4.7±2.9 in 3D ROI. The mean SUV_max_ of PSMA-negative metastases was 1.0±0.5 in 2D and 1.0±0.4 in 3D ROI. In pulmonary opacities, the mean SUV_max_ was 2.2±0.7 in 2D ROI and 2.4±0.8 in 3D ROI. Overall, PSMA-positive lung metastases demonstrated the highest tracer uptake, significantly higher than pulmonary opacities (*p*<0.001). PSMA-negative metastases demonstrated the lowest tracer uptake, significantly lower than pulmonary opacities (*p*<0.001). All pulmonary metastases taken together, there was no difference in mean SUV_max_ between pulmonary metastases and opacities (*p*>0.05). The mean SUV_max_ of PSMA-positive, PSMA-negative and all metastases compared to pulmonary opacities and the aorta is illustrated in Figs. 4 and 5.

**Table 2:** Characteristics of the pulmonary metastases and opacities. Overview of the lesions’ localizations according to lung segments and visual CT morphologies

	Metastasis	Opacity
Number	91	14
Right/Left/Both	54/37/-	4/8/2
Configuration		
Smooth	52	-
Lobulated	7	-
Irregular	32	5
Confluent	-	9
Lung segment		
1	3	1
2	14	1
3	12	0
4	8	1
5	13	2
6	16	1
7	2	0
8	6	0
9	2	1
10	15	5
Multiple	-	2

**Table 3:** Comparison of size, ^68^Ga-PSMA-HBED-CC uptake (SUV_max_) and radiodensity (HU_mean_) between pulmonary metastases (all, PSMA-positive and PSMA-negative) and opacities. PSMA-positive lung metastases demonstrated the highest tracer uptake, significantly higher than pulmonary opacities, whereas PSMA-negative metastases demonstrated the lowest tracer uptake, significantly lower than pulmonary opacities (both *p*<0.001). All pulmonary metastases taken together, the mean SUV_max_ of pulmonary metastases and opacities did not significantly differ (*p*>0.05). *SUV*_*max*_ Maximum standardized uptake value, *HU*_*mean*_ Mean Hounsfield units.

	All pulmonary metastases	PSMA-positive pulmonary metastases	PSMA-negative pulmonary metastases	Pulmonary opacities	*p*-value^a^	Aorta	*p*-value^b^
Diameter in mm	10.96±6.29 (5.2 – 35.8)	11.82±6.98 (5.2 – 35.8)	8.71±3.05 (6.0 – 21.7)		>0.05		
SUV_max_ 2D ROI	3.52±2.78 (0.35 – 13.85)			2.24±0.73 (1.27 – 3.71)	>0.05	1.54±0.34 (0.91 – 2.32)	= 0.001
		4.48±2.68 (1.54 – 13.85)			< 0.001		
			0.99±0.46 (0.35 – 2.13)		< 0.001		
SUV_max_ 3D ROI	3.71±2.96 (0.35 – 15.08)			2.41±0.81 (1.35 – 4.10)	>0.05	1.57±0.34 (0.88 – 2.20)	< 0.001
		4.74±2.86 (1.75 – 15.08)			< 0.001		
			1.01±0.44 (0.35 – 1.96)		< 0.001		
HU_mean_, non-CE CT	-211.92±164.02 (-710.96 – 50.44)	-199.30+-175.84 (-710.96 – 50.44)	-237.16+-138.89 (-632.3 – -67.5)				
HU_mean_, CE CT	-129.78±133.70 (-664.77 – 25.38)	-130.37+-145.70 (-664.77 – 25.38)	-127.44+-74.62 (-220.57 – -2.44)				

Data is given as mean ± standard deviation and range in parentheses. ^a^Metastasis vs. opacity. ^b^Aorta vs. opacity

The mean SUV_max_ obtained by 3D ROI was significantly higher than that obtained by 2D ROI in pulmonary opacities (*p*<0.05) as well as in PSMA-positive lung metastases (*p*<0.001). There was no difference in SUV_max_ of PSMA-negative metastases between 2D and 3D ROI (*p*>0.05).

In all pulmonary metastases taken together, HU_mean_ was -129.8±133.7 in CE-CT and -211.9±164.0 in unenhanced CT. In PSMA-negative metastases, HU_mean_ was -127.4±74.6 in CE-CT and -237.2±138.9 in unenhanced CT; in PSMA-positive metastases, HU_mean_ was -130.4±145.7 in CE-CT and -199.3±175.8 in unenhanced CT.

### Correlation between of size and 3D SUV_max_ of pulmonary metastases

**Fig. 6:**
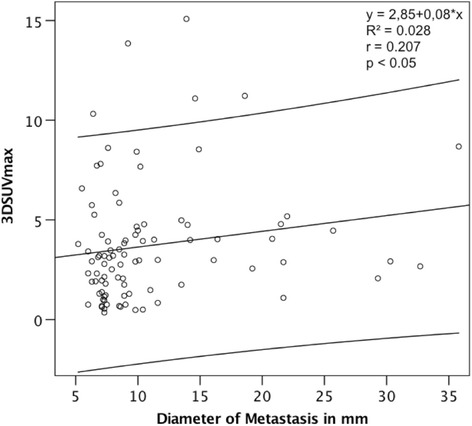
Correlation between size and 3D SUV_max_ of metastases. Linear correlation according to a Spearman’s correlation, including 95% confidence intervals. A weak significant association between the SUV_max_ in pulmonary metastases in 3D regions of interest and their size was calculated (*p*<0.05, ρ_Spearman_=0.207, 95% CI [0.02, 0.396]). *R*^*2*^ Coefficient of determination, *r* Spearman’s rho, *SUV*_*max*_ Maximum standardized uptake value

We calculated a weak, significant positive linear relationship between size and 3D SUV_max_ of metastases (Fig. 6, ρ_Spearman_=0.207, 95% CI [0.002; 0.396], *p*<0.05).

### Patient-based analysis of pulmonary metastases

Of 20 patients with lung metastases, three patients (15%) had ten or more metastases, twelve patients (60%) had two to ten metastases, and five patients (25%) had a single metastasis. Regarding the tracer uptake, twelve patients (60%) had PSMA-positive lung metastases only, seven patients (35%) had mixed metastases, and one patient (5%) had PSMA-negative metastases only.

## Discussion

This study investigated the imaging characteristics of pulmonary metastases and opacities in ^68^Ga-PSMA-PET. Based on the SUV_max_ in ^68^Ga-PSMA-PET alone, it was not possible to differentiate between pulmonary metastases and pulmonary opacities. The majority of lung metastases highly overexpressed PSMA, while a relevant number of metastases were PSMA-negative. Pulmonary opacities demonstrated a moderate tracer uptake, significantly lower than PSMA-positive lung metastases, yet significantly higher than PSMA-negative metastases.

### ^68^Ga-PSMA-PET/CT for the assessment of lung metastases

Our study demonstrates that, although a majority of PC lung metastases were PSMA-positive, a considerable share of metastases was PSMA-negative and could therefore not be detected directly by ^68^Ga-PSMA-PET. Up to now, the largest study investigating the ^68^Ga-PSMA-PET imaging of PC pulmonary metastases and primary lung cancer was conducted by Pyka et al., with 45 patients and 89 lesions [20]. Pyka et al. demonstrated that, due to the high tracer uptake in lung cancer, differentiation between primary lung cancer and lung metastases by SUV analysis was not possible [20]. Within their cohort, mean SUV_max_ of lung metastases was 4.4±3.9, which is consistent with the SUV_max_ calculated in our cohort. Although Pyka et al. did not explicitly differentiate between PSMA-positive and PSMA-negative metastases, they observed a great heterogeneity of tracer uptake in pulmonary metastases; many metastases showed only a faint tracer uptake [20]. This finding is also consistent with our study, in which 27.5% of metastases were PSMA-negative. A possible explanation for the difference of ^68^Ga-PSMA-HBED-CC uptake in lung metastases is the diversity of phenotypes in metastases; especially neuroendocrine trans-differentiation has been identified as a main factor for the loss of PSMA-expression in visceral metastases [26]. This can be explained by the fact that a large part of neuroendocrine prostate cancer cells does not express generic PC biomarkers such as P501S, PSMA, and PSA [27]. Neuroendocrine trans-differentiation was proven histologically in the single patient of our study who had only PSMA-negative pulmonary metastases. A case report by Shetty et al. of a non-PSMA-avid PC lung metastasis suggests that an uncommon variant of the primary PC, in this case ductal adenocarcinoma, can be another cause for missing PSMA-expression [28]. However, the detection rate of the more common lymph node and bone metastases in PSMA-PET appear to be much higher. Schwenck et al. reported detection rates of 94% for lymph node and 98% for bone metastases [29]. Regarding the overall incidence of PSMA-negativity in prostate cancer, Mannweiler et al. found 5% of primary prostate cancer and 15% of prostate cancer metastases to be PSMA-negative in immunohistochemistry [30].

Compared to most other tissues, background tracer uptake of the lungs in ^68^Ga-PSMA-PET is low [31, 32]. Therefore, PSMA-positive lung metastases are clearly visible and detectable as a result of a high lesion-to-background contrast. The low PSMA-expression in the normal lung parenchyma has been confirmed immunohisto-chemically, since bronchioles and terminal bronchioles of normal lung did not stain [33].

Regarding the prevalence in our cohort, lung metastases were present in 2.7% of patients who underwent ^68^Ga-PSMA-PET. This is consistent with the prevalence in imaging of 3.6% reported by Fabozzi et al. [18].

Pulmonary opacities in our study demonstrated a moderate PSMA-expression, not significantly different from pulmonary metastases. There have been some case reports of elevated radiotracer uptake in pulmonary opacities in ^68^Ga-PSMA-PET [22, 31].

It is thought that tracer uptake in pulmonary opacities might not be associated with an increased avidity of the lesion, but due to increased capillary penetration caused by inflammation, which results in a higher tracer activity in the interstitial space [22]. However, another explanation could be the PSMA-expression in the neovasculature of physiologic regenerative and reparative conditions as shown by Gordon et al. [34]. This question could be a subject of investigation for future studies.

A significant positive linear correlation between size and 3D SUV_max_ of PSMA-positive lung metastases was observed. This is likely due to the fact that PSMA-positive metastases tended to be larger than PSMA-negative metastases.

### Limitations

This retrospective study is limited by the fact that diagnoses of pulmonary lesions were not confirmed histopathologically since no biopsies of most of the lesions were performed. Although, to our best knowledge, no patients with secondary malignancy were included, we cannot rule out in each case that solitary lesions considered as metastases were in fact primary pulmonary neoplasms. The study included only lesions > 5 mm in diameter. PSMA-avidity in smaller lesions close to the spatial resolution of the scanner (4.7 mm) could be underestimated due to partial volume effect. However, there was no significant difference between the mean size of PSMA-negative and PSMA-positive lung metastases. Therefore, the variant PSMA-avidity cannot be explained by partial volume effect only.

## Conclusions

Based on the SUV_max_ in ^68^Ga-PSMA-PET alone, it was not possible to differentiate between pulmonary metastases and pulmonary opacities. The majority of lung metastases highly overexpressed PSMA, while a relevant number of metastases were PSMA-negative. Pulmonary opacities demonstrated a moderate tracer uptake, significantly lower than PSMA-positive lung metastases, yet significantly higher than PSMA-negative metastases. Nevertheless, given the combined information of CT scans as well as follow-up scans and the patient’s history, experienced readers should be able to diagnose pulmonary metastases in ^68^Ga-PSMA-PET accurately in most cases.

## Abbreviations

PET/CT: Positron emission tomography / computed tomography
PC: Prostate cancer
Ga: Gallium
PSMA: Prostate specific membrane antigen
SUV: Standardized uptake value
ROI: Region of interest
PSA: Prostate-specific antigen
GS: Gleason score
CE-CT: Contrast-enhanced CT
HU: Hounsfield unit
ADT: Androgen deprivation therapy
RP: Radical prostatectomy
RT: Radiotherapy
CTX: Chemotherapy
